# Supplementary material for: Refined Feasibility Testing of an 8-Week Sport and Physical Activity Intervention in a Rural Middle School
Source: Int J Environ Res Public Health. 2024 Jul 12;21(7):913. doi: 10.3390/ijerph21070913 (PMC11276868; doi:10.3390/ijerph21070913)
Supplement: Supplementary file 1 [file ijerph-21-00913-s001.zip › ijerph-3072890-supplementary.pdf]

Supplementary Material

Table S1. Regression results for physiological and psychological factors.

Variables	Relatedness	Autonomy	Competence	Total BPN
		Total PA		
Control	(0.025, 0.398)	(0.078, 0.51)	(0.071, 0.492)	0.091, 0.541)
Test	(0.088, 0.138)	(0.071, 0.962)	(0.034, 0.489)	(0.008, 0.305)
		Daily Avg. Steps		
Control	(0.071, 0.492)	(0.199, 0.148)	(0.071, 0.492)	(0.096, 0.234)
Test	(0.061, 0.729)	(0.061, 0.729)	(0.018, 0.277)	(0.048, 0.591)
		6MWT		
Control	(0.102, 0.802)	(0.097, 0.668)	(0.094, 0.723)	(0.101, 0.774)
Test	(0.091, 0.106)	(0.027, 0.491)	(0.010, 0.382)	(0.021, 0.246)
		Plank		
Control	(0.097, 0.668)	(0.052, 0.981)	(0.094, 0.723)	(0.110, 0.97)
Test	(0.091, 0.106)	(0.027, 0.491)	(0.053, 0.954)	0.324, 0.005) *

* Adjusted R^2, p-value.
